# Risk factors and predictive model for abdominal wound dehiscence in neonates: a retrospective cohort study

**DOI:** 10.1080/07853890.2021.1938661

**Published:** 2021-06-12

**Authors:** Shouxing Duan, Xuan Zhang, Xuewu Jiang, Wenhui Ou, Maxian Fu, Kaihong Chen, Xinquan Xie, Wenfeng Xiao, Lian Zheng, Shuhua Ma, Jianhong Li

**Affiliations:** a Department of Pediatric Surgery, The First Affiliated Hospital of Shantou University Medical College, Shantou, China; b Department of Pediatric Surgery, The Second Affiliated Hospital of Shantou University Medical College, Shantou, China; c Department of Pediatric Surgery, Shenzhen Pingshan District Woman’s and Children’s Hospital, Southern Medical University, Shenzhen, China; d Department of Radiology, The First Affiliated Hospital of Shantou University Medical College, Shantou, China

**Keywords:** Abdomen wound dehiscence (AWD), neonates, risk factors, hypoproteinemia, incision contamination, surgery

## Abstract

**Background:**

Abdominal wound dehiscence (AWD) is a major complication of abdominal surgery, and neonates are a group with a high risk of AWD, which has serious consequences or can even result in death. The purpose of this study is to explore the risk factors for neonatal AWD and construct a predictive model.

**Methods:**

The clinical data of 453 cases that underwent neonatal laparotomy from June 2009 to June 2020 were retrospectively analyzed, among which 27 cases of AWD were identified. Nine factors, including gender, age at admission, weight at admission, preterm delivery, level of preoperative anaemia, hypoalbuminemia, operation time, incision length, and incision type, were analyzed to explore their correlation with neonatal AWD.

**Results:**

The incidence of neonatal AWD was 6.0% (27/453), among which partial wound dehiscence accounted for 4.9% (22/453) and complete wound dehiscence accounted for 1.1% (5/453). Hypoproteinemia and incision type were the independent risk factors for neonatal AWD, and weight at admission was a protective factor for AWD in the multivariate models. All these factors were incorporated to construct a nomogram, and a calibration curve was plotted. The result indicated that the actual risk was close to the predicted risk when the predicted risk rate was greater than about 35%.

**Conclusions:**

Neonatal AWD is closely related to hypoproteinemia and incision contamination. Our predictive model showed the potential to provide an individualized risk estimate of AWD for neonatal patients undergoing abdominal surgery.

**Key messages**
- Neonatal abdominal wound dehiscence (AWD) has a serious consequence and the incidence of neonatal AWD was about 6.0% and the complete AWD morbidity is 1.1%.
- Hypoproteinemia and incision type were the independent risk factors for neonatal AWD.
- Our predictive model showed the potential to provide an individualized risk estimate of AWD for neonatal patients undergoing abdominal surgery.

## Introduction

With the rapid development of modern neonatal surgery, an increasing number of operations are carried out during the neonatal period, especially abdominal surgeries [1]. Abdominal wound dehiscence (AWD) is a major complication of abdominal surgery, and it is accompanied by high morbidity and mortality in neonates [2]. Because of the unique pathophysiological and anatomical characteristics of newborns, the situation of abdominal wound rupture is more complex and the consequences are more serious, as compared to those in children and adults [3]. The thin abdominal wall of a newborn is more likely to rupture again, with the possibility of causing systemic infection and endangering life [2,4,5]. AWD includes both partial and complete wound dehiscence, partial wound dehiscence means when the skin and/or deep tissue is dehiscent, but the fascia layer is intact, no viscera and omentum can be seen; complete wound dehiscence is considered present when intestine, omentum or other viscera are seen through the abdominal wound. However, there are few reports describing the risk factors for AWD.

Therefore, we sought to investigate the risk factors for neonatal AWD and construct a predictive model, so as to identify effective measures to better prevent and control wound dehiscence.

## Methods

### Study design and patient population

This retrospective observational cohort study consisted of all patients (*n* = 453) who underwent neonatal laparotomy between June 2009 and June 2020, and 27 of these 453 cases developed AWD. All 27 patients with AWD were included. Exclusion criteria were as follows: Discharged without treatment; Patient records were incomplete. The clinical data for all children with AWD were compiled, and they included gender, age at admission, weight at admission, preterm delivery, preoperative anaemia, hypoalbuminemia, operation time, incision length, and incision type. This retrospective study was registered at researchregistry.com (NO.: RR-5350). The study was approved by the Ethics Committee at the Affiliated Hospital of Shantou University Medicine College (NO.: 2016-16) and individual consent for this retrospective analysis was waived.

### Data investigation criteria

AWD includes both partial and complete wound dehiscence. Partial wound dehiscence is a condition characterized by rupture of a surgical incision along the suture so that the skin and/or deep tissue of an abdominal wound are exposed, but the fascial layer is intact and viscera and omentum cannot be seen. Complete wound dehiscence is considered to have occurred when the intestine, omentum or other viscera are seen through the abdominal wound.

In China, a neonate is defined as a baby aged less than 28 days. A preterm infant is defined as a baby born at a gestational age (GA) <37 weeks, and a term infant is defined as a baby born at a GA ≥37 weeks. The hypoproteinemia standard is albumin <35 g/L. Neonatal anaemia is characterized by venous haemoglobin (HB) <130 g/L or peripheral blood HB ≤145 g/L within 2 weeks of birth; after 2 weeks of birth, anaemia is characterized by venous blood HB ≤115 g/L. Neonatal anaemia is classified as follows: if the infant’s age is less than or equal to 2 weeks and the HB level is between 100 and 145 g/L, the condition is classified as mild to moderate anaemia; if the HB level is less than 100 g/L, it is classified as severe anaemia. If the infant’s age is more than 2 weeks and the HB level is between 80 and 115 g/L, it is classified as mild to moderate anaemia; if the HB level is less than 80 g/L, it is considered to be severe anaemia. Surgical incision type can be divided into the following three categories: type I incision (clean incision): sterile incision that can be sutured; type II incision (possibly contaminated incision): suture incision that may be contaminated during surgery; and type III incision (contaminated incision): refers to the adjacent infected area or tissue directly exposed to the infection [6].

### Management after AWD

The abdominal wounds were examined from the third postoperative day onwards on a daily basis to identify the signs of wound infection, dehiscence including redness (erythema), seroma formation, discharge of serous fluid or pus from one or more sites and subsequently partial or complete wound dehiscence.

The patients with partial wound dehiscence were managed conservatively by laying open the wound, and daily wound washing and dressing along with intravenous antibiotics according to culture and sensitivity. It can be cured by conservative treatment, but complications, such as incisional hernia, could occur in some patients.

In cases of complete wound dehiscence, the wound was immediately covered with sterile cotton pads to protect the bulge and fissure once the intestinal and/or omental prolapse were visible. At the same time, the patients were sedated to avoid an increase in abdominal pressure and release more intestinal tubes caused by continuous crying. The patients were transferred to the operating room for extended suturing under general anaesthesia. During surgery, the intestine was washed out, any residual suture in the wound was removed, the necrotic tissue was removed, and an entire layer of decompression suture was added. The application of antibiotics and local dressing changes were strengthened after the operation. The sutures were removed 2 to 3 weeks later. Table 1 presents the intraoperative findings and treatment of cases with complete wound dehiscence.

**Table 1.** The intraoperative findings and treatment of complete wound dehiscence cases.

Case	Disease	Condition during the first operation	The first operation	Wound orientation	Dehiscence after surgery (d)	Condition during the second operation	The second operation	Length of stay (d)
1	Congenital intestinal atresia	Type II atresia, approximately 40 cm from the flexion ligament	Bowel resection and anastomosis	Horizontal	5	No anastomotic fistula	Debridement and tension suture	29
2	Ileal perforation	An idiopathic perforation was located at approximately 50 cm from the ileocecal area	Bowel repair	Vertical	8	Intestinal repair healed well	Debridement and tension suture	37
3	Meconium peritonitis	Intestinal adhesions were severe during surgery, and fistulization was made 35 cm from the ileocecal area	Enterostomy	Vertical	6	Unexplored abdominal cavity	Debridement and tension suture + stoma replacement	23
4	Congenital intestinal malrotation	Bowel rotation 270° clockwise	Ladd operation and appendectomy	Horizontal	5	The thread that ligated the appendix became untied, and pus was seen in the abdominal cavity, but there was no fistula of the appendix	Abdominal lavage + debridement and tension suture	27
5	Neonatal necrotizing enterocolitis with perforation	Multiple perforations of the colon and fistulization was made 10 cm from the ileocecal area	Enterostomy	Vertical	7	Unexplored abdominal cavity	Debridement and tension suture + stoma replacement	74

### Statistical analysis

SPSS software 22.0 (IBM, USA) was used for statistical analysis. Categorical variables were expressed as frequencies and percentages. Categorical variables were compared using the Chi-squared test or Fisher’s exact test, as appropriate. Univariate and multivariate binary logistic regression analyses were conducted to identify independent predictors of AWD, and the odds ratio (OR) and 95% confidence intervals (CI) were computed using binary logistic regression analysis. A nomogram and calibration curve were established using R software (version 4.0.4) and Rstudio (version 1.1.383). The level of significance was set at *p*＜.05.

## Results

Among the 453 patients included in this study, 27 cases developed AWD. These 27 infants were 1–28 days old, and they weighed 1.9–3.5 kg. There were 17 males and 10 females, and 19 term infants and 8 preterm infants. The patient demographics are shown in Table 2.

**Table 2.** Patient demographics.

Parameter	Data (number)
Sex	Males (298); females (155)
Gestational age (week)	38.51 ± 1.59
Age of admission (d)	7.79 ± 8.40 (1–28)
Bodyweight (kg)	2.80 ± 0.51 (1.9–3.8)
Wound orientation	Horizontal (337)
	Vertical (116)
AWD types	Partial wound dehiscence (22)
	Complete wound dehiscence (5)
Etiology	Congenital intestinal atresia/stenosis (117)
	Intestinal perforation (51)
	Meconium peritonitis (12)
	Congenital intestinal malrotation/twist (105)
	Congenital hypertrophic pyloric stenosis (62)
	Congenital defects of gastric musculature (12)
	Mesenteric/omental cyst (16)
	Neonatal necrotizing enterocolitis with perforation (23)
	residual diseases of omphalomesenteric duct (55)

In this study, AWD occurred 3–8 days after the initial surgery. The incidence of AWD was 6% (27/453), among which partial wound dehiscence accounted for 4.9% (22/453) and complete wound dehiscence accounted for 1.1% (5/453). The patients with partial wound dehiscence were cured by conservative treatment, the 5 cases with complete dehiscence were cured by debridement and tension suture, and there was no recurrence of dehiscence or death. The patients were followed up for 6 to 12 months, and the wounds healed well.

### Analysis of risk factors

**Figure 1.**
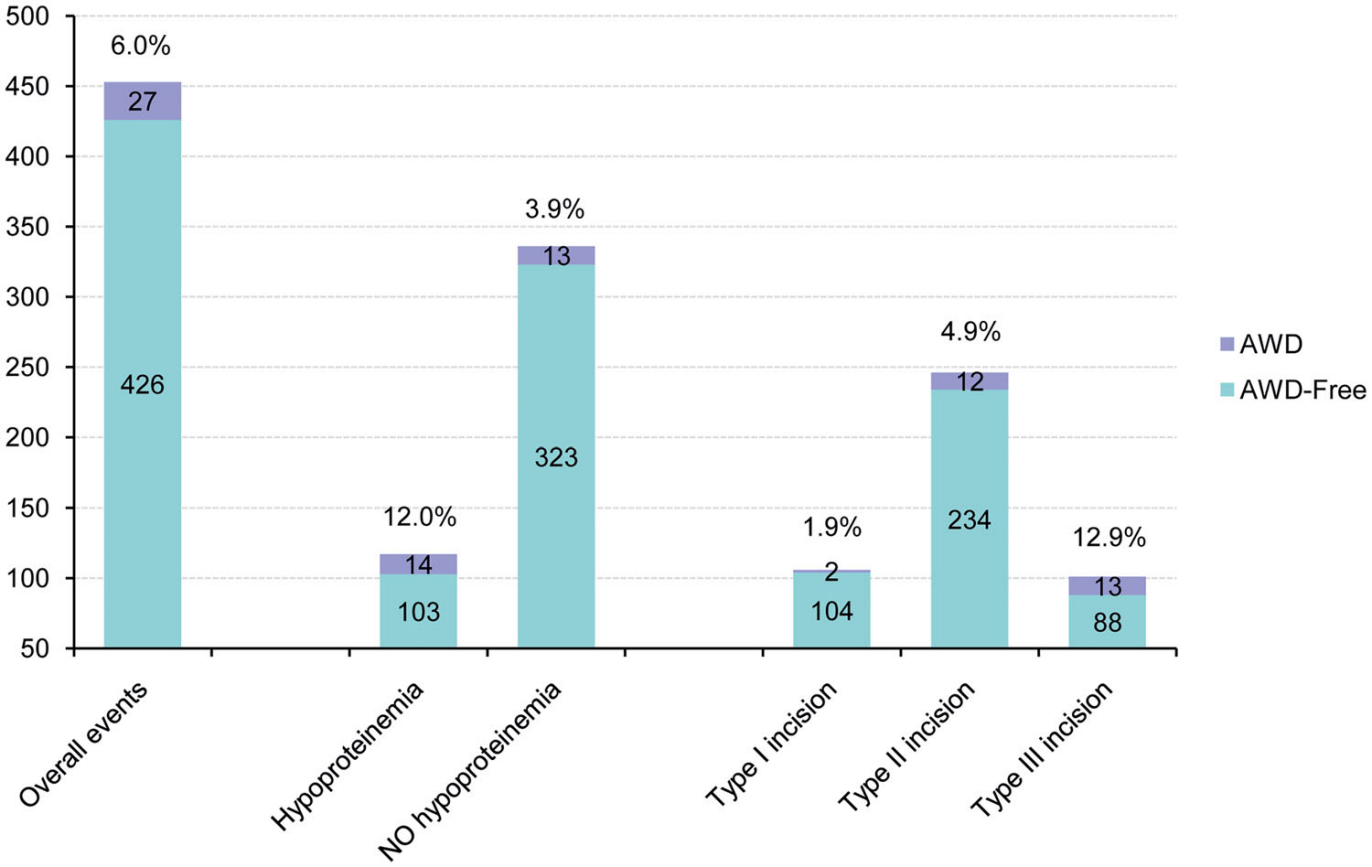
Clinical outcomes in patients with independent risk factors and the overall events.

After univariate analysis of risk factors, we found that gender, age at admission, weight at admission, preterm delivery, operation time, and incision length were not the risk factors for AWD, and the difference was not statistically significant (*p* > .05); however, the level of preoperative anaemia, hypoproteinemia, and incision type were the potential risk factors for AWD (*p* < .05) (Table 3). Significant variables after univariate correlation analysis were further analysed by binary logistic regression, and the results of multivariate stepwise logistic regression analysis indicated that hypoproteinemia (OR 2.841, 95% CI 1.249–6.460) and incision type (OR 2.274, 95%CI 1.256–4.118) were independently associated with the presence of the composite endpoint (Table 4). Hypoproteinemia and incision type were the independent risk factors for neonatal AWD. The incidence of independent risk factors associated with AWD and the overall events are shown in Figure 1.

**Table 3.** Correlation analysis of risk factors for neonatal AWD.

Factors	Total cases (*N* = 453)	AWD		Chi-squared value	*p*-value
		(*N* = 27)	Rate (%)		
Gender					
Male	298	17	63.0	0.101	.750
Female	155	10	37.0		
Admission age					
<7 days	283	14	51.9	1.381	.240
7–28 days	170	13	48.1		
Admission weight					
<2500g	113	8	29.6	0.337	.562
≥2500g	340	19	70.4		
Preterm delivery					
Premature infant	54	4	14.8	0.229	.632
Term infant	399	23	85.2		
Preoperative anaemia					
Severe anaemia	30	5	18.5	8.216	.004
Mild and moderate anaemia	68	6	22.2		
No anaemia	355	16	59.3		
Hypoproteinemia					
Preoperative hypoproteinemia	117	14	51.9	10.150	.001
No hypoproteinemia	336	13	48.1		
Operation time					
<2 h	337	19	70.4	0.244	.621
≥2 h	116	8	29.6		
Incision length					
<5 cm	155	9	33.3	0.010	.921
≥5 cm	298	18	66.7		
Incision type					
I	106	2	7.4	12.259	.002
II	246	12	44.4		
III	101	13	48.1		

**Table 4.** Significant variables after binary logistic regression analysis.

Variable	Wald	Odds ratio	95% CI	*p*-value
Hypoproteinemia	6.206	2.841	1.249–6.460	.013
Incision type	7.350	2.274	1.256–4.118	.007

### Nomogram construction and validation

**Figure 2.**
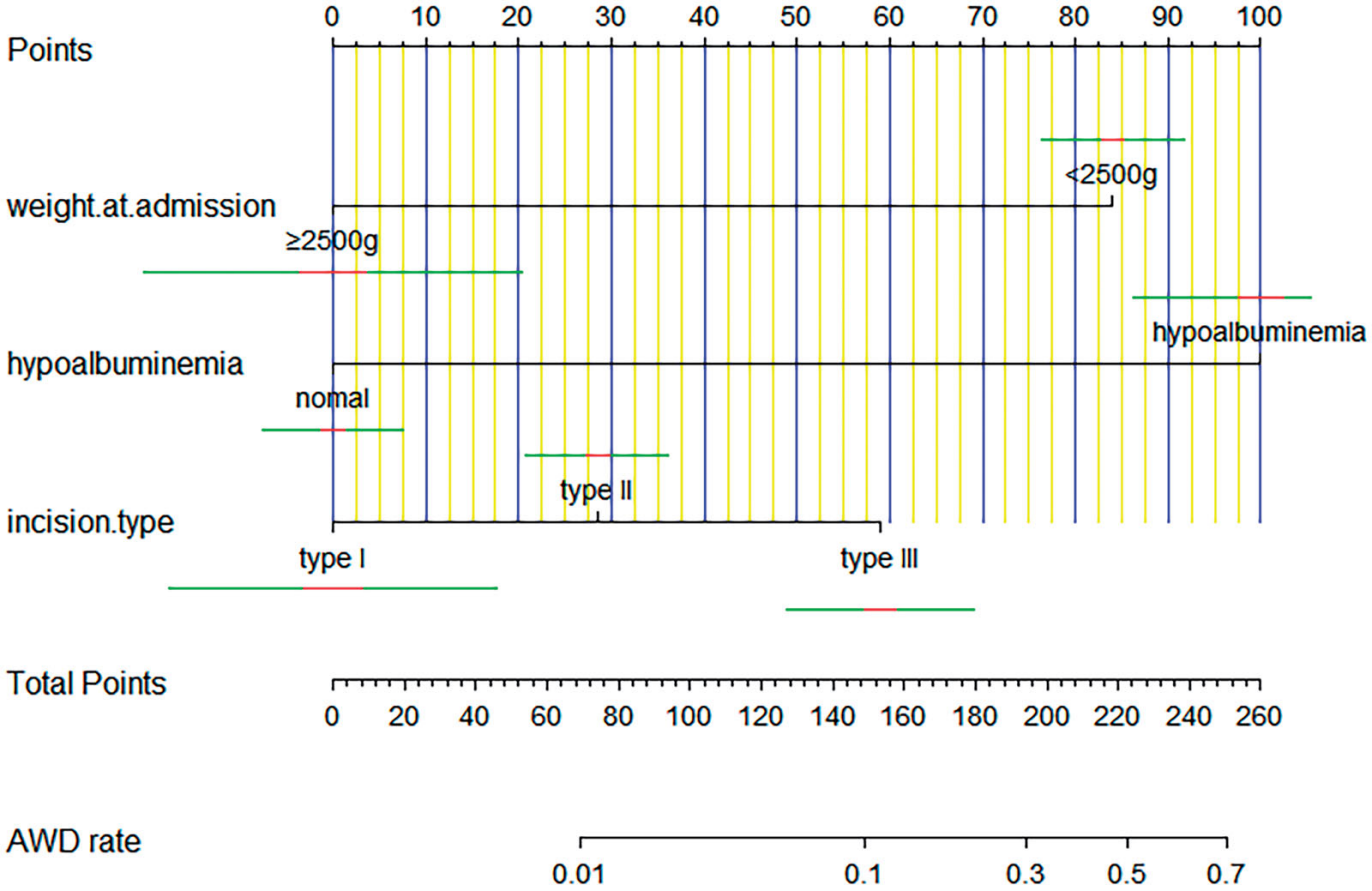
Nomogram for predicting AWD rate. The red and green indicate the confidence interval.

**Figure 3.**
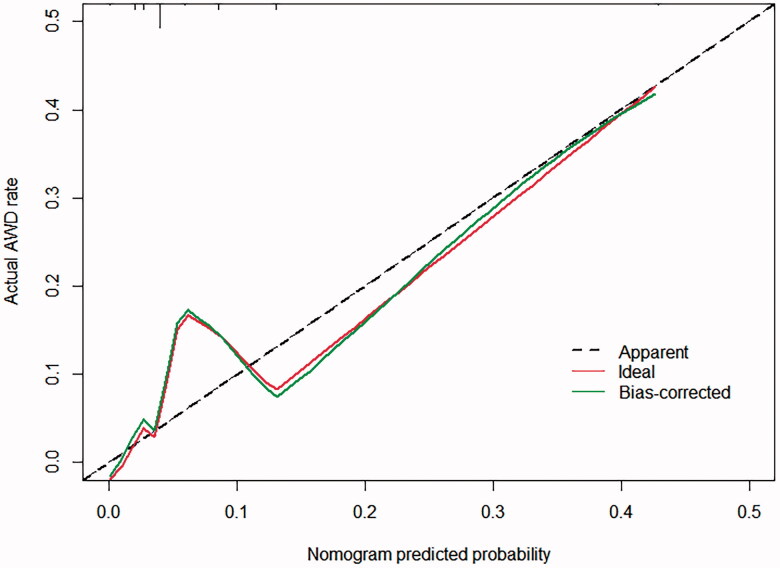
AWD nomogram calibration curve. The plot shows the relationship between the actual probability and predicted probability.

Multivariate analyses demonstrated that the variables, such as hypoproteinemia and incision type, were independent risk factors for AWD, but the weight at admission (OR 0.081, 95% CI 0.020–0.324) was a protective factor for AWD. A nomogram including all significant independent factors for predicting AWD was established (Figure 2). Each variable assigned a score on a point scale; by adding the scores of each selected variable, we could easily estimate the probability of AWD in an individual patient. The C-index for the nomogram to predict AWD was 0.760 (95% CI, 0.668–0.851). Then we performed calibration of the nomogram internally with bootstrap sampling 1000 times and a calibration curve (Figure 3) was plotted, and it showed the relationship between the actual probability and predicted probability. When the predicted risk rate was greater than about 35%, the baseline and the reference line basically coincided, and the actual risk was close to the predicted risk.

## Discussion

With the development of modern medical approaches and anaesthesia, there has been a significant increase in the number of neonatal abdominal surgical operations in some developing countries due to the recognition and detection of neonatal gastrointestinal malformations [1,7]. Because of the immature development of organ function in neonates, low immune function and rapid change in disease are more prominent. Newborns are a high-risk group for AWD compared to older children and adults [2,8]. Wound dehiscence is the result of multiple factors, and it usually occurs within 1 week after surgery [9,10]. In the current study, 9 factors, including gender, age at admission, weight at admission, preterm delivery, level of preoperative anaemia, hypoalbuminemia, operation time, incision length, and incision type were analyzed to explore their relationship with neonatal AWD.

In the current study, we found that the incidence of neonatal AWD was 6.0% (27/453) and that AWD occurred 3 to 8 days after surgery, mostly within 7 days. Through the analysis of the above-related factors, we found that hypoalbuminemia and incision type were the independent risk factors for neonatal AWD.

It has been reported that the incidence of wound dehiscence after infection is 5–10 times as high as that after primary healing [11]. It has been noted that infection is an important cause of wound dehiscence. In the group of neonates included in the current study, contaminated incisions (types II and III) accounted for 92.6% (25/27) of AWD cases. The chances of contamination of the surgical field are higher than in aseptic surgery. The incidence of incision dehiscence increases with an increase in the degree of contamination. All 5 cases of complete wound dehiscence had undergone intestinal tract surgery, and all of them received more than a type II incision. In 2 cases of enterostomy, the stoma was placed at the original wound made during the first surgery, and the intestinal fluid contaminated the wound and caused infection; in 1 case of congenital intestinal malrotation, the second abdominal exploration showed that the thread used to ligate the appendix became loose, and pus was found in the abdominal cavity; and in the other 2 patients, the abdominal cavity was contaminated by faeces. Contamination occurred in the above-mentioned patients, indicating that wound contamination is an important risk factor for AWD. There was a positive correlation between the incision type and AWD, and the incidence increased with the aggravation of contamination.

Malnutrition is an important factor affecting postoperative recovery [12,13]. Albumin is the most commonly used index to evaluate nutritional status, and hypoproteinemia is closely related to the surgical outcome and postoperative complications [13–15]. In our study, we found that the incidence of AWD in patients with preoperative hypoproteinemia was 12%, while the incidence of AWD in patients without preoperative hypoproteinemia was only 3.9%; the former percentage was 3 times more than the latter percentage. The results indicated that preoperative hypoproteinemia is closely related to AWD because the condition is not conducive to the healing of an abdominal wound. Healing becomes hindered because hypoproteinemia can reduce the synthesis of collagen and collagenase and the growth of granulation tissue on the wound surface, resulting in poor wound healing [16]. In patients with hypoproteinemia, the colloid osmotic pressure is low, and wound exudation is increased, which provides a medium for the growth and reproduction of bacteria and causes poor wound healing [17]. In addition, hypoproteinemia will lead to the hypofunction of several system organs, especially immune function, causing a decrease in the ability of the injured skin to protect against infection, which can lead to wound infection and dehiscence [18,19].

Vigilance is required to prevent neonatal AWD during the entire perioperative period [20,21]. Because of the lack of nutritional reserve and the poor tolerance of surgery by neonates, perioperative nutritional support should be strengthened to improve the surgical success rate [22,23]. The presence of anaemia or electrolyte disorders should be corrected before surgery, and for those infants who are not able to accept oral liquid nourishment after surgery, total venous nutrition and albumin should be used to provide positive nitrogen balance [23,24]. In addition to adequate washing in patients who have a severe abdominal cavity infection, effective antibiotics should also be used for the control of infection [25]. In patients who develop fistulization, another incision can be created for stoma replacement to prevent the first wound from being contaminated by the intestinal fluid. In patients with multiple risk factors, a preventive tension suture can be used [26,27].

Hypoproteinemia and incision type were identified as the independent risk factors for neonatal AWD in our study, but the weight at admission was a protective factor for AWD in the multivariate models. We established a nomogram including all significant independent factors for predicting AWD, from which the probability of AWD in an individual patient can be easily estimated. Likewise, we plotted a calibration curve that can show the relationship between the actual probability and predicted probability. Our predictive model showed the potential to provide an individualized risk estimate of AWD for neonatal patients undergoing abdominal surgery. It could provide satisfactory predictions when the predicted risk was greater than 35%.

## Conclusions

In conclusion, our predictive model has the potential to provide an individualized risk estimate of AWD for neonatal patients undergoing abdominal surgery. Hypoproteinemia and incision type were the independent risk factors for the occurrence of neonatal AWD. Therefore, it is very important to clean the abdominal cavity and incision thoroughly during the operation and strengthen the nutritional support and infection control after the operation in the high-risk population of neonates.

## Data Availability

The datasets generated in this study are available from the corresponding author on reasonable request.

## References

[1] Chirdan LB, Ngiloi PJ, Elhalaby EA. Neonatal surgery in Africa. Semin Pediatr Surg. 2012;21:151–159. 22475121 10.1053/j.sempedsurg.2012.01.007

[2] Duan SX, Wang GH, Zhong J, et al. Clinical analysis of entire abdominal wound disruption in neonates. J Clin Ped Sur. 2017;16:601–603,16.

[3] Jensen KK, Oma E, van Ramshorst GH, et al. Abdominal wound dehiscence is dangerous: a nationwide study of 14,169 patients undergoing elective open resection for colonic cancer. Hernia. 2021;[online 2021 Jan 4]. DOI:10.1007/s10029-020-02350-z 33394254

[4] Jiang XW, Chen ZX, Lai YM, et al. Neonatal and infants abdominal wall incision dehiscence clinical features. J Appl Clin Pediatr. 2002;17:265–266.

[5] Di Saverio S, Tarasconi A, Walczak DA, et al. Classification, prevention and management of entero-atmospheric fistula: a state-of-the-art review. Langenbeck’s Archives of Surgery. 2016;401:1–13. 10.1007/s00423-015-1370-3 26867939

[6] Chen XP, Wang JP. Surgery. 8th ed. Beijing: People’s Sanitary Publishing Press; 2013; p. 103–104.

[7] Duan SX, Sun ZB, Wang GH, et al. Diagnosis and treatment of pediatric benign pneumoperitoneum: a case report series of 9 patients. Medicine. 2017;96:e5814. 28079808 10.1097/MD.0000000000005814 PMC5266170

[8] Visscher MO, Adam R, Brink S, et al. Newborn infant skin: physiology, development, and care. Clin Dermatol. 2015;33:271–280. 25889127 10.1016/j.clindermatol.2014.12.003

[9] Geng XP, Sun J. Prevention and treatment of incision dehiscence. Chin J Prac Surg. 2007;27:45–47.

[10] Denys A, Monbailliu T, Allaeys M, et al. Management of abdominal wound dehiscence: update of the literature and meta-analysis. Hernia. 2021;25(2): 449–462. 32897452 10.1007/s10029-020-02294-4

[11] Heller L, Levin SL, Butler CE. Management of abdominal wound dehiscence using vacuum assisted closure in patients with compromised healing. Am J Surg. 2006;191:165–172. 16442940 10.1016/j.amjsurg.2005.09.003

[12] Weimann A, Braga M, Carli F, et al. ESPEN guideline: clinical nutrition in surgery. Clin Nutr. 2017;36:623–650. 28385477 10.1016/j.clnu.2017.02.013

[13] Takagi K, Domagala P, Hartog H, et al. Current evidence of nutritional therapy in pancreatoduodenectomy: systematic review of randomized controlled trials. Ann Gastroenterol Surg. 2019;3:620–629. 31788650 10.1002/ags3.12287 PMC6875945

[14] Marcason W. Should albumin and prealbumin be used as indicators for malnutrition? J Acad Nutr Diet. 2017;117:1144. 28648265 10.1016/j.jand.2017.04.018

[15] Shin KH, Han SB. Early postoperative hypoalbuminemia is a risk factor for postoperative acute kidney injury following hip fracture surgery. Injury. 2018;49(8):1572–1576. 29908852 10.1016/j.injury.2018.05.001

[16] Gundogdu RH, Yasar U, Ersoy PE, et al. Effects of preoperative nutritional support on colonic anastomotic healing in malnourished rats. Ulus Cerrahi Derg. 2015;31:113–117. 26504412 10.5152/UCD.2015.3077 PMC4605104

[17] Wang J, Zhao M, Liang R, et al. Whey peptides improve wound healing following caesarean section in rats. Br J Nutr. 2010;104:1621–1627. 20691127 10.1017/S0007114510002692

[18] Kim J, Shim SH, Oh IK, et al. Preoperative hypoalbuminemia is a risk factor for 30-day morbidity after gynecological malignancy surgery. Obstet Gynecol Sci. 2015;58(5):359–367. 26430660 10.5468/ogs.2015.58.5.359 PMC4588840

[19] Raiten DJ, Sakr Ashour FA, Ross AC, et al. Inflammation and nutritional science for programs/policies and interpretation of research evidence (INSPIRE). J Nutr. 2015;145:1039s–1108s. 25833893 10.3945/jn.114.194571 PMC4448820

[20] Childs DR, Murthy AS. Overview of wound healing and management. Surg Clin North Am. 2017;97:189–207. 27894427 10.1016/j.suc.2016.08.013

[21] Jovanovic G, Jakovljevic DK, Lukic-Sarkanovic M. Enhanced recovery in surgical intensive care: a review. Front Med. 2018;5:256. 10.3389/fmed.2018.00256 PMC6180254 30338259

[22] Zhao P, Liu X, Zuo W, et al. Clinical analysis of 55 cases of neonatal digestive tract perforation. J Clin Ped Sur. 2011;10:470–471.

[23] Cai W, Tang QY. Emphasis on the importance of perioperative nutritional support in critically ill patients. Chin J Pediatr Surg. 2014;33:201–202.

[24] Di Saverio S, Tarasconi A, Inaba K, et al. Open abdomen with concomitant enteroatmospheric fistula: attempt to rationalize the approach to a surgical nightmare and proposal of a clinical algorithm. J Am Coll Surg. 2015;220:e23–33. 25537306 10.1016/j.jamcollsurg.2014.11.020

[25] Yu H, Ge YL. Rational application of antibiotics in special children. Chin J Appl Clin Pediatr. 2013;8:721–723.

[26] Krishnan R, MacNeil SD, Malvankar-Mehta MS. Comparing sutures versus staples for skin closure after orthopaedic surgery: systematic review and meta-analysis. BMJ Open. 2016;6:e009257. 10.1136/bmjopen-2015-009257 PMC4735308 26792213

[27] Jategaonkar PA, Yadav SP. Modified intradermal ‘figure-of-eight’ suture for cosmetic closure of umbilical port site incision. Ann R Coll Surg Engl. 2014;96:388–389. 24992427 10.1308/rcsann.2014.96.5.388 PMC4473940

